# Intradermal insulin infusion achieves faster insulin action than subcutaneous infusion for 3-day wear

**DOI:** 10.1007/s13346-015-0239-x

**Published:** 2015-06-03

**Authors:** Christopher James Rini, Elaine McVey, Diane Sutter, Stephen Keith, Heinz-Joerg Kurth, Leszek Nosek, Christoph Kapitza, Kerstin Rebrin, Laurence Hirsch, Ronald J. Pettis

**Affiliations:** BD Technologies, Durham, NC USA; Profil, Neuss, Germany; BD Medical - Diabetes Care, Franklin Lakes, NJ USA

**Keywords:** Glucose, Glycemia, Insulin, Microneedle, Clinical research

## Abstract

Rapid uptake previously demonstrated by intradermal (ID) drug administration indicates compound delivery within the dermis may have clinical and pharmacological advantages for certain drug therapies. This study is the first clinical trial to evaluate continuous microneedle-based drug infusion, device wearability, and intradermal microneedle insulin kinetics over a multi-day (72 h) wear period. This was a single center, open-label, two-period crossover study in T1DM patients on continuous subcutaneous insulin infusion (CSII). Patients received treatment during interventional visits: one SC and one ID basal/bolus infusion of insulin aspart (NovoRapid® U-100) administered over 3 days in a randomized order. Twenty-eight patients were randomized and exposed to trial product, and 23 completed the study. Bolus insulin infusions were given prior to standardized breakfast and lunch test meals on each of the three treatment days. Blood samples were drawn at predefined time points for measurements of insulin aspart and blood glucose in serum. The primary endpoint insulin Tmax demonstrated that ID bolus infusion was associated with a significantly shorter Tmax with statistically significantly smaller intra-subject variability, compared to SC infusion, and this difference was maintained over three treatment days. Analyses of secondary PK endpoints corresponded with the primary endpoint findings. Postprandial glycemic response was significantly less pronounced after ID bolus: For most endpoints ID vs. SC, differences were statistically significant within the 0–1.5 or 0–2 h time period. Intradermal delivery of insulin is a viable delivery route alternative providing reduced time for insulin absorption with less intra-subject variability and lower glycemic response.

## Introduction

Faster insulin action to mimic pancreatic response to blood glucose (BG) is a fundamental challenge for improved glycemic control in diabetes patients. Postprandial glycemic excursions in healthy individuals are believed to be small (i.e., in the range of 20–50 mg/dL above baseline glycemia) and return to normal range within 2 h despite consuming considerable amounts of carbohydrates with meals [1, 2]. In order to mimic the physiologic rapid increase in insulinemia, which also rapidly decreases hepatic glucose production [3], rapid-acting insulin analogs have been developed [4]. Current rapid-acting insulin analogs have faster subcutaneous (SC) kinetics than regular insulin, but do not replicate the normal pancreatic insulin secretion [5]. Delayed and unpredictable insulin uptake creates challenges for managing postprandial glycemic excursions and establishing predictive control for closed-loop insulin delivery systems.

Traditional SC injection or infusion delivers insulin into the subcutaneous adipose tissue, from which it is absorbed into the bloodstream. Continuous subcutaneous insulin infusion (CSII) is an effective therapy to minimize hypoglycemia and maintain long-term glycemic control in type I diabetes patients. Pump therapy use is expanding among type 1 diabetes patients as new and improved pump technologies emerge. With the recognition that pump therapy will be an enabling part of a closed-loop artificial pancreas system, all components of the delivery system, including the pump and infusion set, must be critically evaluated, validated, and optimized for delivery in order to maintain the appropriate and anticipated insulin dosing schedule for maintenance of blood glucose within a normal range. Within this context, researchers are continually investigating the efficacy, safety, and patient acceptability for alternative formulations [6, 7], devices [8], and routes of insulin delivery (oral [9], transdermal [10], inhaled [11], intra-nasal [12], and intradermal [13, 14]) in efforts to enhance glycemic control.

**Fig. 1:**
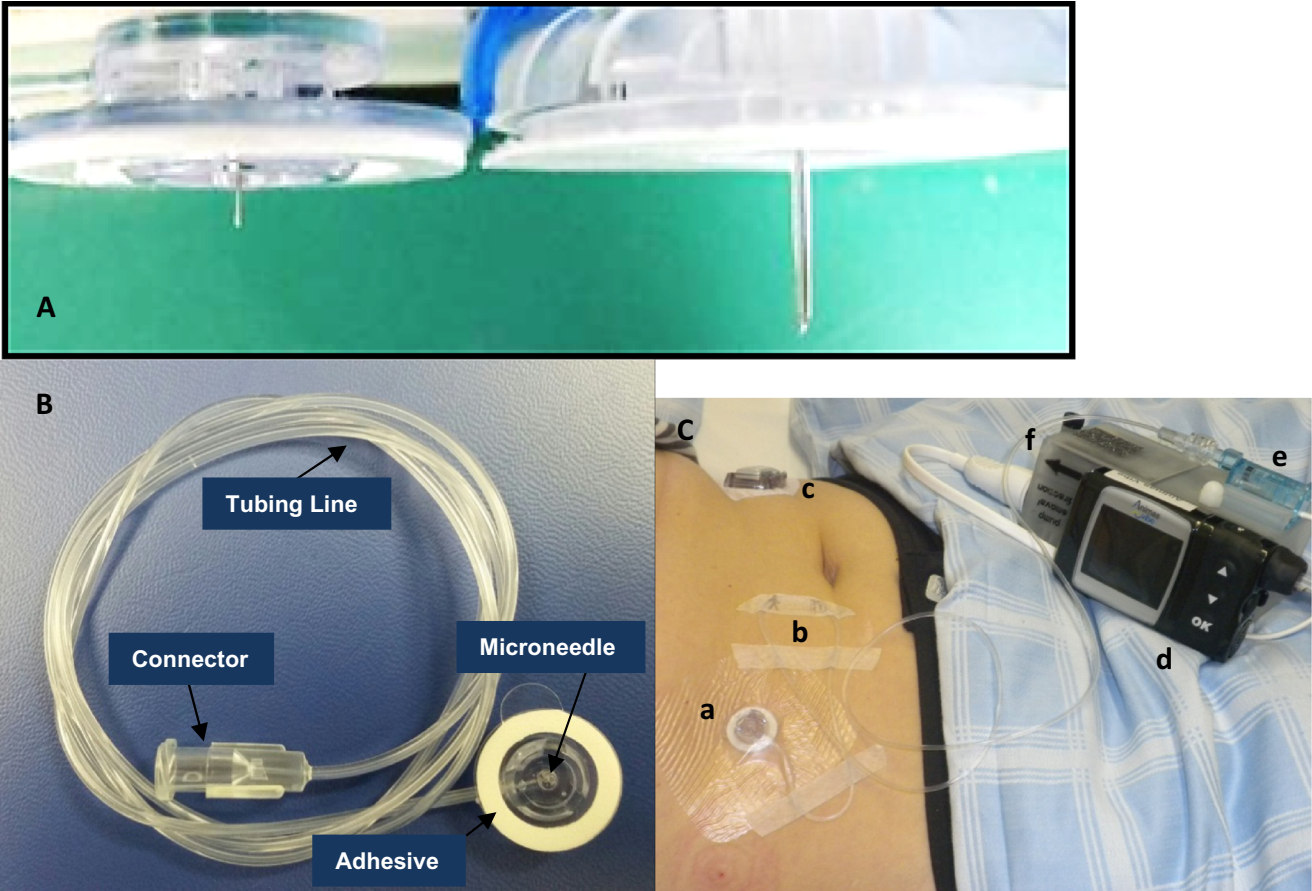
**a** Investigational microneedle infusion set with a 34G × 1.5 mm stainless steel microneedle (*left*) compared to a standard 25G × 6 mm polymer cannula on a commercial CSII set. **b** Investigational intradermal microneedle catheter set showing components: 1.5-mm microneedle, fluid tubing line, skin adhesive, and Luer connector for joining to insulin infusion pumps. **c** ID microneedle catheter with IV3000 overtape on a patient’s abdomen (*a*) showing tubing safety loops (*b*), CGM for BG safety monitoring (*c*), insulin pump (*d*), in-line pressure transducer (*e*), and infusion pressure monitoring hardware (*f*)

There is no standardized definition or dimensional specification for microneedles, and various systems have been utilized for targeted administration to the epidermal and dermal tissue layers as a means to bypass the physical and chemical transport barriers imposed by the stratum corneum. A variety of fabrication techniques, designs, materials, and usage methods have been employed to create diverse devices including solid microneedles to enhance passive epidermal transport, coated or dissolvable solid microneedles for in situ dissolution, and hollow microneedles for direct fluid transport to the dermis [15, 16]. In this study, a single hollow stainless steel microneedle (34 G × 1.5 mm length; 178 and 63.5 µm nominal outer and inner diameters) was utilized for direct administration of clinically meaningful basal and bolus insulin doses to the dermal tissue bed over a 3-day infusion period (Fig. 1a, b). The device incorporated traditional design and functional aspects of commercial SC infusion sets including application method and tubing line connections for commercial CSII pumps. The microneedle catheter set used herein has been extensively characterized in preclinical [17] and clinical studies to ensure consistent and effective intradermal administration for insulin and other drugs [18, 19].

Intradermal (ID) injections are used clinically for the delivery of some vaccines such as flu, rabies, cholera, and Bacille Calmette-Guérin (BCG) for tuberculosis, some local medications, such as lidocaine for local anesthesia, lymph node staging [20], and some diagnostic tests, such as the Mantoux test to assess immune status against tuberculosis [13]. However, delivery of drug substances, such as insulin, into the dermis is not routine. Rapid systemic uptake and increased bioavailability have been previously demonstrated in preclinical animal models using single hollow stainless steel microneedles for ID delivery of protein therapeutics [17], indicating that delivery of compounds within the dermis may have clinical and pharmacological advantages for certain drug therapies [21]. A potential rationale for these altered pharmacokinetic effects include enhanced vascular uptake from dense dermal capillary beds. Alternatively, human clinical studies indicate that rapid uptake may be driven by a lymphatic mechanism that transports insulin from the skin administration site via draining lymph vessels and into the systemic circulation [13].

Previously reported clinical studies have demonstrated viable ID microneedle delivery up to 24 h with placebo solutions [22], continuous ID basal insulin infusion for up to 6 h [18, 19], and faster insulin action from bolus ID insulin lispro delivery compared to the standard subcutaneous route [23–27]. This study is the first clinical trial to evaluate continuous microneedle-based drug infusion including both basal and bolus administration, device wearability, and intradermal microneedle insulin kinetics over a multi-day (72 h) wear period. Additionally, this is the first ID clinical use of the analog insulin aspart.

## Materials and methods

### Patients

Male and female type 1 diabetes mellitus (T1DM) patients in general good health on insulin pump therapy with carbohydrate counting for at least 6 months, aged 18 to 55 years, with BMI ≤ 32 kg/m^2^, with HbA1c ≤ 8.0 %, using ≤60 U of insulin on a typical day, and negative tests for hepatitis B/C and HIV at screening, were eligible for participation in the study. Exclusion criteria included conditions that could interfere with insulin absorption (lipodystrophy), distribution (impaired cardiac function, uncontrolled hypertension), clearance (gastroparesis, impaired hepatic, or renal functions), and other concomitant interfering medications or conditions as judged by the investigator.

### Study design and procedures

This was a single center, open-label, two-period crossover study, randomized by infusion order, in T1DM patients on continuous subcutaneous insulin infusion (CSII). The protocol was approved by an independent ethics committee (Ethikkommission der Ärztekammer Nordrhein, Düsseldorf, Germany), appropriate regulatory bodies (German Federal Institute for Drugs and Medical Devices (BfArM)), and conducted in compliance with good clinical practice (GCP), the Declaration of Helsinki, and applicable regulatory requirements.

The study was completed over a multi-week period consisting of three study visits: a screening visit (V1) and two interventional visits (V2 and V3). After establishing eligibility and enrollment, patients received treatment during two interventional visits: one SC and one ID basal/bolus infusion of insulin aspart (NovoRapid® U-100, Novo Nordisk, Copenhagen, Denmark) administered over 3 days in a randomized order.

For each dosing visit, patients arrived the evening prior to the first standardized meal to verify compliance with protocol criteria. An intravenous catheter was inserted into a peripheral vein of the arm for manual blood sampling. Infusion sets, ID (34 G × 1.5 mm length stainless steel microneedle catheter set, BD Technologies, Research Triangle Park, NC) or SC (25 G × 6 mm length soft cannula, Quick-Set, Medtronic Minimed, Northridge, CA), were connected to an insulin infusion pump (Animas® Vibe™, Animas Corporation, West Chester, PA) and primed according to the manufacturer’s instructions. Sets were placed into the patient’s abdomen (periumbilical, left or right) using a customized applicator for the ID set, or the manufacturer recommended inserter for the SC set (customized Sof-serter® and Quick-serter® respectively, both Medtronic Minimed, Northridge, CA). Due to the small adhesive surface on the microneedle catheter, a modified IV dressing (IV3000 1-Hand, Smith & Nephew, England) was used for additional securement over the catheter. A safety loop was used on all catheter lines as recommended by general insulin pump user guidance (Fig. 1c). The respective infusion sets provided all basal and bolus insulin delivery of insulin aspart for the duration of each 72-h study period. A fluid pressure transducer (BD-DTX™ Plus, Becton Dickinson, Franklin Lakes, NJ) was connected in-line between the pump reservoir and each infusion set to monitor infusion delivery pressure, with automated data capture via a commercial data logging system (LORD MicroStrain®, Williston, VT) to provide a secondary post hoc measure of delivery status. The pump/pressure monitoring system combination was contained in a waist pack for the duration of delivery due to its potentially cumbersome size.

Individual meal bolus doses were determined based on patient self-reported insulin to carbohydrate ratios and insulin sensitivity, historical self-reported insulin usage for meal coverage, and clinician review and adjustment of insulin usage during the in-clinic BG stabilization period the evening prior to the first morning meal. Basal insulin infusion rates were initiated based on the patient’s known daily delivery profile and could be adjusted by the clinician to maintain levels within 70–160 mg/dL according to a predetermined sliding scale and hourly changes in glycemia. A continuous glucose monitor (Seven Plus®, DexCom Inc., San Diego, CA) calibrated per manufacturer’s recommendations was applied to continuously monitor glycemia as a safety measure, in addition to periodic blood sampling for BG determination.

**Fig. 2:**
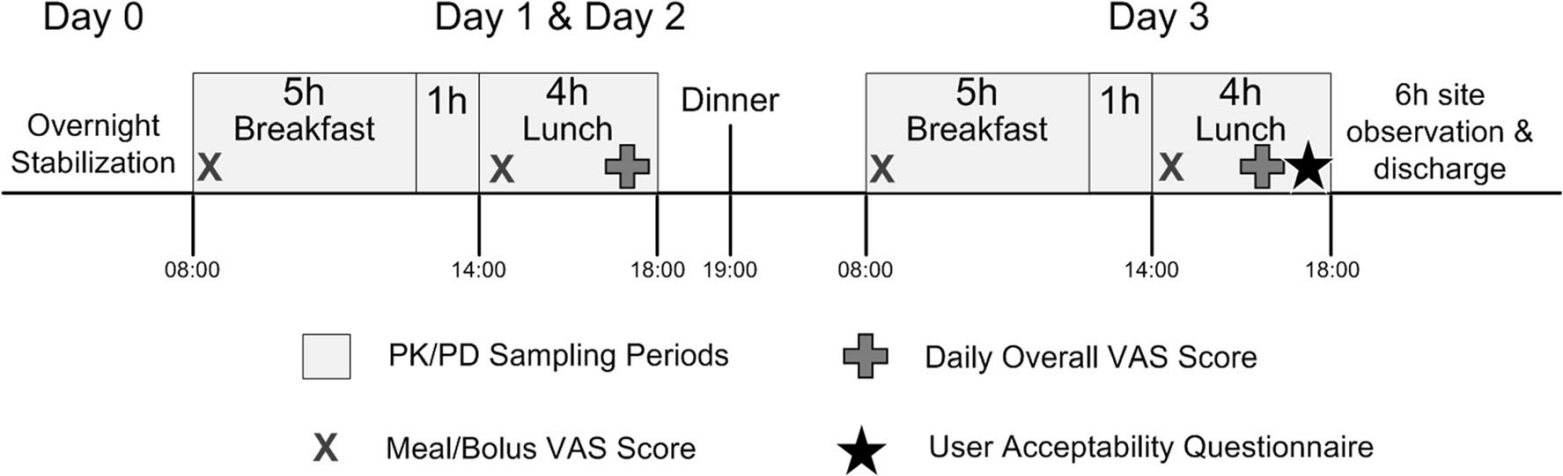
Timeline and procedures for interventional dosing days, including standardized breakfast and lunch meals with PK/PD sampling, user ratings, and questionnaire

After maintaining targeted blood glucose range during the preceding night, bolus insulin doses were administered via the respective infusion catheter prior to standardized breakfast and lunch test meals on each of the three treatment days. Each meal insulin bolus was calculated based on the patient’s insulin sensitivity and previous hours change in BG per sliding scale then delivered immediately prior to breakfast (*t* = 0 min) and lunch (*t* = 360 min) consumption. Breakfast was a high glycemic index (60 g carbohydrates) solid meal, and lunch was a mixed meal based on the individual’s calculated daily energy intake and equivalent in caloric composition by day. Breakfast and lunch meals were consumed within 15 min followed by serial blood sampling with BG monitoring for a period of 6 and 4 h, respectively. Patients were asked to remain sedentary for 2 h after meals. Identical study procedures were utilized for the remaining dosing visit days (Fig. 2).

### PK/PD assessments

To assess insulin absorption kinetics and glycemic response, blood samples for measurements of insulin aspart and blood glucose in serum were drawn at predefined time points throughout dosing visits. Serial samples for safety BG determination, but not PK analysis, were obtained at a minimum every 2 h. Meal PK/PD analysis samples were acquired immediately pre-bolus and for 4–6 h post-bolus for breakfast and lunch meals, with a higher frequency during the first 2 h to capture insulin and BG peak levels {Post-bolus breakfast time points (min): 0, 10, 15, 20, 25, 30, 35, 40, 45, 50, 55, 60, 65, 70, 80, 90, 120, 150, 180, 210, 240, 300, 330, 360; Post-bolus lunch time points (min): 0, 10, 20, 30, 40, 50, 60, 70, 80, 90, 120, 180, 240, 300}. Sampling times were based on previous clinical study experience for bolus microneedle insulin delivery and had increased sampling frequency in the first 90 min post-delivery to capture the rapid insulin peaks from microneedle ID delivery [18, 19]. Blood samples for evening meal and basal period PK assessment could not be obtained due to daily blood draw volume limits for patient safety. Serum insulin aspart was measured by a third party laboratory (IKFE GmbH, Mainz, Germany) using a validated insulin assay (Invitron ELISA kit). The ELISA kit was not aspart specific; however, serum insulin concentrations were baseline corrected by subtracting the calculated mean serum insulin concentration obtained from measurements at 30, 15, and 0 min before meal intake from all subsequent values. Blood glucose levels for PD assessments were measured in-clinic using a standard laboratory method (Super GL Ambulance glucose analyzer, Freital, Germany).

### Study endpoints

The primary PK endpoint was evaluating time and delivery route (ID vs. SC) effects after a meal bolus excursion on insulin Tmax across the multi-day dosing period, with the study design powered to this endpoint. Secondary PK endpoints included maximum plasma insulin concentration (Cmax), time to 50 % Cmax during insulin onset (T50%max rising) and offset (T50%max falling), area under the plasma insulin versus time curve (AUC insulin), and intra- and inter-subject PK variability. PK endpoints after evening meals and during basal periods could not be measured due to blood volume sampling limitations and so were not included for analysis. PD values were used for subject safety monitoring throughout the study period; however, the data analysis set comprised only the 10-h period after the breakfast meal. Postprandial PD secondary endpoints included area under the BG versus time curve (AUC BG), absolute BG, change in BG (ΔBG), maximum BG (BGmax), average BG (BGavg), and change in BGmax (ΔBGmax). In order to provide a complete picture of insulin absorption and effect, PK and PD endpoints were evaluated at several time points (1, 1.5, 2, 4 h) or time periods (0–1, 0–1.5, 0–2, and 0–4 h). Device performance and lifetime was evaluated by monitoring adhesion to treatment site, leakage, pump occlusion alarms, and changes in insulin PK data over time.

### Perception and safety

Safety evaluations comprised of recording/classification of adverse events (including hypoglycemia, BG < 60 mg/dL with or without symptoms), physical examinations, clinical laboratory parameters, vital signs, subjective pain assessments, and assessments of local tissue response at the injection site. Visual observations of the area around the infusion site for skin irritation, insulin leakage, and any notable abnormalities were recorded after each bolus delivery. Upon device removal, the study physician checked the site of insulin administration, as well as 1 and 2 h later, and scored any local reactions according to the Draize erythema and edema scale.

Patients rated bolus-associated pain immediately after insulin delivery for both meals and again each evening at approximately 6 PM after completion of the morning dosing and sampling cycles to rate overall discomfort from the infusion process during the preceding 24 h. The visual analog scale (VAS) utilized a standard validated 10-cm scale anchored by “no pain” (score 0) and “severe pain” (score 10) without inclusion of additional numerical or verbal descriptors to avoid clustering of scores around intermediate points. Scores were measured as absolute distant from origin. Within 1 h prior to device removal, patients completed a non-validated user acceptability questionnaire consisting of a set of standard multiple choice closed-ended questions covering acceptability of sensations for infusion sets application and operation, set satisfaction, willingness to use the respective infusion set again, and set preference. Responses were restricted to a 5-point scale generally ranging from extreme agreement/satisfaction to extreme disagreement/satisfaction depending upon the question. ID acceptability questions were posed based on perception alone and within the context of having a potentially increased glycemic control benefit. These outcomes were observational in nature, since the study was not powered for perception and acceptability endpoints.

### Statistical analysis

Pharmacokinetic parameters were derived from baseline corrected blood insulin data. The pharmacokinetic and pharmacodynamic populations included all patients with data from either route across 3 days of infusion and patients exposed to at least one trial product with available data respective of PK/PD endpoints. PK and PD endpoints were analyzed using a linear mixed effects model (ANOVA) with sequence, application route, meal, and day as factors along with interactions. The safety population included all patients who had participated in any trial-related activity after randomization. VAS pain data comparisons were made between application routes at each assessment time point using a similar model. Safety evaluations were summarized by descriptive statistics.

## Results

### Patients and demographics

In total, 43 patients were screened and 28 (*n* = 15 men, 13 women) were included and randomized to treatment. All patients were Caucasian. Twenty-three (23) patients completed the study according to the protocol with five additional partial or incomplete data sets. Reasons for incompletion included consent withdrawal (2), insulin delivery failure or pump occlusion alarm during overnight stabilization period (2), and nausea/emesis adverse event occurrence (1). All 28 patients exposed to the investigational product were included in the safety analysis and also constituted the PK/PD analysis set. Mean (±SD) age was 42.14 ± 9.06 years, weight 75.52 ± 10.36 kg, BMI 25.05 ± 2.82 kg/m^2^, and HbA1c 7.16 ± 0.65 %.

### Dosing data

**Fig. 3:**
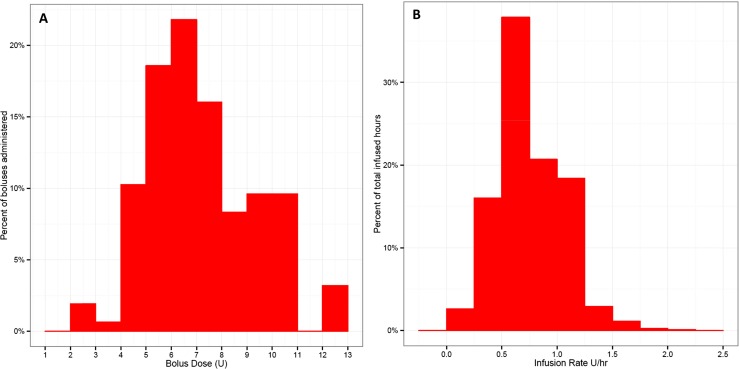
Distribution histogram across all patients (*n* = 28) of **a** ID bolus volumes (mean 6.8 ± 2.1; range 2–12 U) and **b** ID basal rates (mean 0.7 ± 0.3; range 0.1–2.0 U/h) administered during the 3-day infusion. Individual patient boli were dose matched in the SC delivery arm. Basal insulin delivery rates required for euglycemia varied both intra- and inter-patient based on insulin sensitivity and diurnal variation

ID microneedle boli across all analyzed lunch and dinner deliveries (*n* = 156) had a mean (±SD) bolus volume of 6.8 ± 2.1 U insulin (68 ± 21 µl; range 2.0–12.0 U) and were dose matched in the SC delivery arm for an individual patient (Fig. 3a). The insulin pump used in this study delivered boli rapidly in 1 U increments at approximately 2 s/U for both the ID and SC routes. Across this small volumetric range of boli used in the study, any modest differences in dose delivery timing were ignored during subsequent PK analysis. Basal insulin delivery rates varied both between patients and within day for a given patient based on diurnal variability and changing background insulin need to maintain euglycemia. The majority of basal rates were between 0.25–1.25 U/h (2.5–12.5 µl/hour; mean 0.72 ± 0.29 U/h; range 0.1–2.0 U/h) (Fig. 3b), and during the breakfast and lunch bolus periods, background basal rates remained fixed. Detectable pre-bolus basal insulin levels were typically near or below the assay detection limits; however, bolus insulin concentration data was background adjusted based on the average of the three sequential time points (−30, −15, 0 min) prior to the breakfast bolus dosing. Likewise, PK dosing data were analyzed as both non-normalized concentration data and also dose-normalized for individual patient mass and dosing volume (data not shown). Both methods resulted in the same detectable statistical differences for PK endpoints.

### Device performance endpoints

ID delivery throughout the 3-day infusion course was effective with only one noted device adhesive performance issue of partial nonadherence and one detectable incidence of leakage. No other adherence or leakage events for ID or SC devices were recorded.

### PK endpoints

**Fig. 4:**
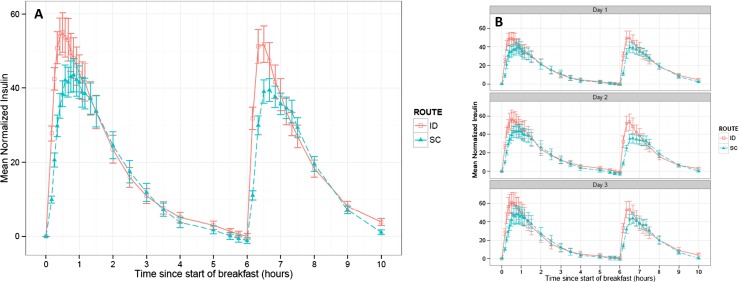
Serum insulin aspart concentration vs. time PK profiles after standardized breakfast and lunch meals across all dosing days. **a** Mean baseline-adjusted serum insulin concentration (mU/L) by route and **b** by treatment day. The data represent mean values (±1SEM) from all 28 patients exposed to trial product

The mean insulin concentration-time profiles across days and per day across meals both illustrate faster onset and offset of insulin action for ID delivery with corresponding increased peak concentrations and are consistent with prior preclinical and clinical studies [17, 23–27] (Fig. 4, Table 1). The primary endpoint of insulin Tmax demonstrated that ID bolus infusion was associated with a statistically significantly shorter (*p* < 0.0001) Tmax (Table 1). The 20-min difference in time to peak concentration represents greater than 40 % reduction compared to the respective SC delivery. This relative difference was also maintained across all three treatment days (Table 1) and both meal types. Similarly, T50_max_rising and T50_max_falling decreased by ~50 and 25 %, respectively (*p* < 0.0001), not only confirming the increased uptake rate but also representing faster insulin offset. Due to the faster uptake, early phase insulin exposure, represented by increased insulin AUC (ΔAUCIns), was also statistically different (between the ID and SC routes at early time points (*p* < 0.0001) and for up to 1.5 h post-bolus). However, these detectable differences resolved by 2 h (NS difference) indicating a shift in the concentration-time profile rather than an increase in total overall insulin bioavailability. Mean overall insulin exposure was routinely lower after the lunch meal owing to lower carbohydrate content with reduced insulin bolus dosing. Although time factors for each dosing route and across meal types were consistent across days, a general trend for increasing maximum insulin concentration (Cmax) and overall ΔAUCIns was noticed across the 3-day dosing period. This time effect was significant (*p* < 0.05) for both endpoints when controlled for route and meal effects but was only between days 1 and 3. Consecutive days (d1–d2 and d2–d3) were not statistically distinguishable in insulin concentration effects.

**Table 1:** Mean postprandial pharmacokinetic parameters for 3-day ID and SC infusion of insulin aspart across treatment days/meals and for individual treatment days (*n* = 141–156)

Parameter	Route	Days 1–3 combined (mean ± SD)	*p* value	Day 1	Day 2	Day 3
Primary endpoint						
Tmax [min]	ID	28.5 ± 13.2	<0.0001	27.6 ± 13.9	29.1 ± 13.3	29.1 ± 12.5
	SC	47.7 ± 22.8		46.0 ± 28.4	50.1 ± 19.7	47.1 ± 18.3
Secondary endpoints						
T50_max_rising [min]	ID	6.0 ± 2.4	<0.0001	5.7 ± 2.2	5.8 ± 2.6	6.4 ± 2.3
	SC	12.2 ± 7.7		13.2 ± 7.9	12.0 ± 9.5	11.2 ± 5.0
T50_max_falling [min]	ID	107 ± 36.1	<0.0001	108 ± 39.2	106 ± 32.6	107 ± 36.7
	SC	138 ± 45.1		135 ± 51.1	143 ± 44.1	136 ± 39.5
ΔCmax [mU/L]	ID	57.9 ± 46.5	0.0121	54.5 ± 42.1	58.6 ± 47.0	61.2 ± 51.5
	SC	48.7 ± 37.6		47.4 ± 37.5	47.2 ± 34.8	51.5 ± 40.8
ΔAUCIns_0−1_ [mU*h/L]	ID	42.5 ± 34.7	<0.0001	39.5 ± 31.4	43.7 ± 36	44.8 ± 37.4
	SC	31.3 ± 24		29.9 ± 25.3	30.6 ± 21.1	33.6 ± 25.7
ΔAUCIns_0–1.5_ [mU*h/L]	ID	60.2 ± 51.6	0.0039	56.6 ± 46.4	61.6 ± 54.0	62.8 ± 55.6
	SC	49.0 ± 39.0		45.9 ± 38.9	48.7 ± 35.9	52.8 ± 42.5
ΔAUCIns_0–2_ [mU*h/L]	ID	72.5 ± 65.5	NS	68.5 ± 59.0	74.0 ± 67.8	75.7 ± 71.2
	SC	62.2 ± 52.3		57.8 ± 50.9	62.6 ± 49.6	66.7 ± 57.0
ΔAUCIns_0–4_ [mU*h/L]	ID	91.0 ± 93.5	NS	86.9 ± 85.8	92.3 ± 94.5	94.4 ± 103
	SC	83.4 ± 81.3		77.4 ± 76.2	85.7 ± 82.3	87.7 ± 86.9

Significant difference *p* < 0.05

*NS* not significant

ID intra-subject Tmax variability measured by %CV was statistically significantly lower vs. SC administration (30 vs. 37 %, *p* = 0.0128) across meals and days (Table 2), consistent with previous bolus meal trials. However, inter-subject variability was not significantly different. Likewise, intra- and inter-subject variability was not significantly different between the two delivery routes for other PK secondary endpoints.

**Table 2:** Intra-subject variability (%CV: percentage coefficient of variation) across all study days and standardized breakfast and lunch meals

Parameter	Time [h]	ID %CV	SC %CV	*p* value
PK				
Tmax [min]		29.6	36.7	0.0128
PD				
BG [mg/dL]	1	29.1	20.2	0.0004
	1.5	31.5	24.5	0.0069
BGavg [mg/dL]	0–1	26.3	18.2	0.0001
	0–1.5	25.6	18.2	<0.0001
	0–2	25.9	19.3	0.0003
BGmax [mg/dL]	0–1	25.2	18.1	0.0004
	0–1.5	24.5	17.9	0.0001
	0–2	23.8	18.5	0.0021
	0–4	21.1	18.0	0.0478
ΔBG [mg/dL]	2	90.2	61.4	0.0347

Nonsignificant variability PK/PD endpoints and time periods have been omitted from table

Fisher’s LSD test; significance indicated by *p* value <0.05

### PD endpoints

**Fig. 5:**
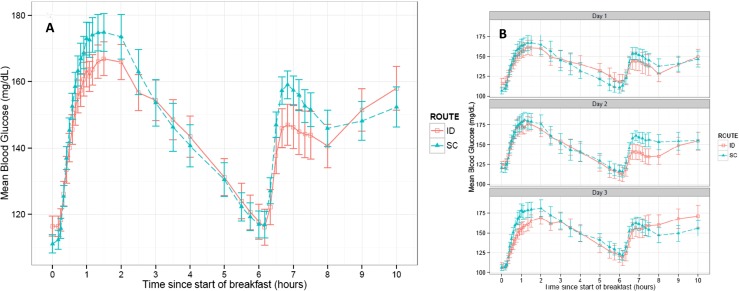
Blood glucose vs. time PD profiles after standardized breakfast and lunch meals across all dosing days. Mean blood glucose (mg/dL) profile by **a** route and **b** by treatment day. The data represent mean values (±1SEM) from all 28 patients exposed to trial product

BG vs. time PD profiles indicate ID insulin administration provides a delay in postprandial rise of glucose levels and reduction in peak glucose excursions (Fig. 5). Intradermal microneedle delivery produced a significant reduction in postprandial glycemic response for multiple PD factors (BGmax, ΔBGmax, ΔAUC-BG) up to two hours after meal consumption (Table 3). Mean blood glucose levels for ID delivery were below SC for both breakfast and lunch meals from ~30–150 and 120 min, respectively. Average blood glucose levels (BGavg, 0–1 h) were not statistically distinguishable within the first hour, and this is attributed to the rapid gastric glucose absorption and distribution independent of route of insulin administration since significant decreases were observed for the BGavg during later time intervals (0–1.5 and 0–2 h). Significant differences between ID and SC routes were reported for BG (at 1 h, 154 versus 165, *p* = 0.0121) and ΔBG in the 0–1 h (*p* < 0.0001) and 0–1.5 h (*p* = 0.0149) time intervals. Intra- and inter-subject variability was not significantly different between the two routes for ΔBGmax and ΔAUC-BG. In contrast to PK parameters, there were statistical differences of some glycemic response parameters for ID infusion indicating slightly higher intra-subject variability (Table 2), whereas the inter-subject variability was not significantly different between the two routes.

**Table 3:** Mean ± SD pharmacodynamic parameters for all test meals across treatment days and for individual days across meals (*n* = 150–156)

Parameter	Route	Days 1–3 combined (mean ± SD)	*p* value	Day 1	Day 2	Day 3
ΔAUC-BG_0–1_ [mg*h/dL]	ID	18.1 ± 21.2	<0.0001	17.2 ± 22.3	18.7 ± 22.0	18.5 ± 19.2
	SC	27.5 ± 22.2		29.1 ± 25.7	26.7 ± 20.9	26.5 ± 19.5
ΔAUC-BG_0–1.5_ [mg*h/dL]	ID	36.0 ± 39.8	<0.0001	32.4 ± 41.7	36.8 ± 40.8	39.3 ± 36.8
	SC	51.9 ± 42.5		53.4 ± 46.7	50.8 ± 40.9	51.3 ± 39.8
ΔAUC-BG_0–2_ [mg*h/dL]	ID	54.5 ± 60.6	0.0004	47.8 ± 63.1	53.9 ± 61.9	62.8 ± 56.3
	SC	75.3 ± 66.5		77.0 ± 73.2	73.6 ± 63.3	75.1 ± 63.0
ΔAUC-BG_0–4_ [mg*h/dL]	ID	126 ± 144	NS	105 ± 148	120 ± 140	156 ± 140
	SC	142 ± 155		133 ± 160	140 ± 157	154 ± 148
BG_1_ [mg/dL]	ID	154 ± 49.3	0.0121	151 ± 56.0	155 ± 45.8	156 ± 45.2
	SC	165 ± 42.1		158 ± 43.8	169 ± 40.9	170 ± 40.9
BG_1.5_ [mg/dL]	ID	154 ± 51.8	NS	146 ± 52.2	155 ± 51.4	163 ± 51.3
	SC	163 ± 49.3		156 ± 51.2	167 ± 48.3	167 ± 48.3
BG_2_ [mg/dL]	ID	154 ± 52.7	NS	145 ± 54.5	152 ± 51.9	165 ± 50.5
	SC	160 ± 55.7		152 ± 60.1	164 ± 54.2	164 ± 52.3
BG_4_ [mg/dL]	ID	150 ± 55.9	NS	145 ± 52.1	147 ± 55.5	160 ± 60.1
	SC	146 ± 53.7		139 ± 52.4	148 ± 59.4	153 ± 48.9
BGavg_0–1_ [mg/dL]	ID	136 ± 40.5	NS	135 ± 45.9	138 ± 37.4	136 ± 37.3
	SC	143 ± 32.2		138 ± 33.4	147 ± 32.5	145 ± 30.3
BGavg_0–1.5_ [mg/dL]	ID	141 ± 40.6	0.0204	137 ± 42.1	144 ± 39.9	143 ± 40.2
	SC	150 ± 35.0		144 ± 36.0	154 ± 35.3	152 ± 33.4
BGavg_0–2_ [mg/dL]	ID	144 ± 42.4	0.0375	139 ± 43.8	146 ± 41.9	148 ± 41.8
	SC	153 ± 38.0		147 ± 39.3	157 ± 38.2	155 ± 36.1
BGavg_0–4_ [mg/dL]	ID	148 ± 43.6	NS	141 ± 44.0	148 ± 41.3	156 ± 45.0
	SC	151 ± 42.0		142 ± 41.2	155 ± 44.0	156 ± 40.1
ΔBGmax_0–1_ [mg/dL]	ID	42.8 ± 30.1	<0.0001	41.3 ± 32.1	43.1 ± 30.2	44.2 ± 28.1
	SC	56.7 ± 33.3		57.1 ± 36.0	55.8 ± 31.0	57.2 ± 33.0
ΔBGmax_0–1.5_ [mg/dL]	ID	49.5 ± 34.1	<0.0001	45.0 ± 34.5	49.5 ± 33.5	54.8 ± 34.2
	SC	63.6 ± 37.6		65.0 ± 42.3	61.6 ± 33.2	63.9 ± 36.8
ΔBGmax_0–2_ [mg/dL]	ID	53.5 ± 35.2	0.0002	49.4 ± 36.7	52.2 ± 33.7	59.5 ± 34.9
	SC	67.8 ± 41.8		69.7 ± 49.0	65.5 ± 34.7	68.1 ± 40.3
ΔBGmax_0–4_ [mg/dL]	ID	67.8 ± 40.8	NS	64.0 ± 43.6	69.4 ± 36.8	70.4 ± 42.2
	SC	75.0 ± 45.6		76.5 ± 54.6	72.3 ± 40.0	76.2 ± 40.4
BGmax_0–1_ [mg/dL]	ID	161 ± 46.2	0.0102	159 ± 53.1	162 ± 43.0	162 ± 41.5
	SC	172 ± 39.2		166 ± 40.1	176 ± 39.2	175 ± 37.9
BGmax_0–1.5_ [mg/dL]	ID	167 ± 45.8	0.0059	160 ± 46.8	169 ± 45.6	172 ± 45.0
	SC	179 ± 41.8		174 ± 43.6	182 ± 41.7	182 ± 40.0
BGmax_0–2_ [mg/dL]	ID	170 ± 46.4	0.0095	165 ± 47.8	171 ± 46.5	176 ± 44.9
	SC	183 ± 45.1		178 ± 49.1	186 ± 43.4	186 ± 42.4
BGmax_0–4_ [mg/dL]	ID	184 ± 47.4	NS	178 ± 49.1	186 ± 43.2	188 ± 49.8
	SC	188 ± 45.4		179 ± 44.4	193 ± 49.3	194 ± 41.7
ΔBG_1_ [mg/dL]	ID	35.6 ± 35.5	<0.0001	33.3 ± 38.2	35.8 ± 35.4	38.2 ± 32.9
	SC	49.8 ± 39.0		49.0 ± 41.5	48.8 ± 37.9	51.8 ± 37.9
ΔBG_1.5_ [mg/dL]	ID	37.2 ± 43.6	0.0149	31.0 ± 44.4	35.8 ± 44.4	45.7 ± 41.2
	SC	47.9 ± 48.2		47.5 ± 51.9	47.2 ± 44.8	48.9 ± 48.1
ΔBG_2_ [mg/dL]	ID	36.7 ± 46.5	NS	30.2 ± 48.5	33.1 ± 46.1	48.0 ± 43.3
	SC	44.6 ± 55.6		43.7 ± 62.7	44.0 ± 51.2	46.2 ± 52.4
ΔBG_4_ [mg/dL]	ID	33.3 ± 54.9	NS	29.3 ± 54.1	28.0 ± 55.7	43.6 ± 54.8
	SC	30.8 ± 52.3		29.7 ± 53.3	27.7 ± 56.8	35.1 ± 47.2

Significant difference *p* < 0.05

*NS* not significant

### Safety endpoints

Assessment of infusion site tissue effects was completed using Draize dermal erythema and edema scoring immediately after removal of the infusion set on day 3 and for several hours thereafter. Upon removal, both routes were equivalent with essentially no detectable erythema or edema. In the subsequent 2 h, both routes exhibited an unexpected increase in both edema and erythema. Erythema severity was similar between routes, with the majority of sites ≤1, but several instances (4/24 patients) with Draize 2 for ID route. Edema scores were also predominantly ≤1 for both ID and SC delivery; however, three scores of Draize 2 and single instances each of Draize scores 3 and 4 for the ID route were observed 2 h after removal. All sites were reported as normal by the 6 h time point. Draize scoring was not blinded to the observer and represented the composite reaction to both the delivery process and dermal reaction to medical adhesives and long-term dressing occlusion. Notwithstanding this, the frequency and severity of transient dermal effects was modestly elevated for the ID route.

No clinically relevant changes were observed for clinical laboratory tests, vital signs, or physical examinations in study patients. In total, 31 adverse events (AEs) of mild to moderate severity were reported during the entire study, with none recorded as serious. Headache was reported most frequently with 13 cases in 6 subjects, followed by dizziness (8 cases, 4 subjects), nausea (3 cases, 3 subjects), and vomiting (2 cases, 2 subjects). The other reported AEs were only single occurrences. Of these events, 3 were noted as probably, 13 as possibly, and 15 as unlikely treatment related, and all were resolved. There was neither indication of any bias in the number or type of AEs between treatment route nor evidence of safety concerns for ID compared to SC delivery.

In total, 123 detected hypoglycemic episodes, defined as single or consecutive BG measurements below 60 mg/dL, occurred across the study with the majority being asymptomatic. For ID infusion, 67 episodes occurred in 22 subjects, with 56 episodes in 18 subjects after SC infusion. Six hypoglycemic episodes occurred prior to any bolus delivery, and only 11 episodes were associated symptomatic and associated with other documented AE. All were of mild severity, short duration (≤1.2 h), and all were resolved, with the majority treated by oral administration of orange juice/snack. The observed number of asymptomatic hypoglycemic episodes may have been influenced by both the intense BG safety monitoring as well as the tight control exercised in the clinical environment.

### Perception endpoints

**Fig. 6:**
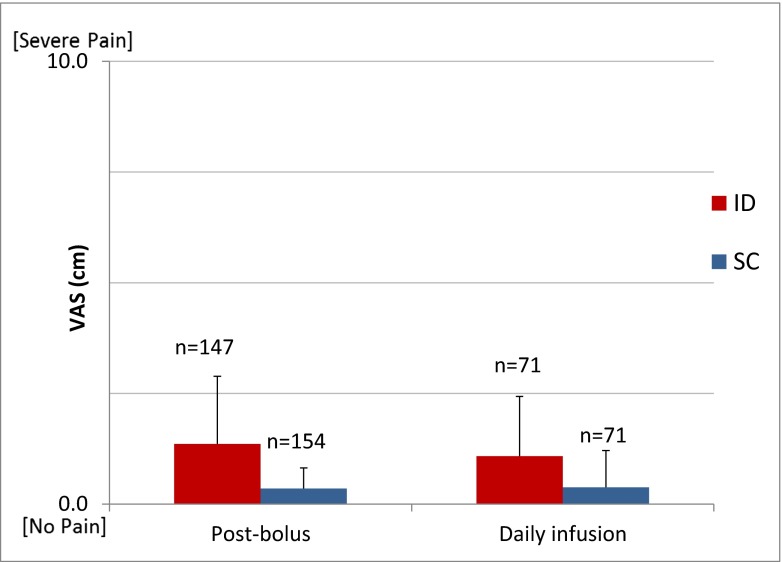
Mean (± SD) VAS self-rated pain perception immediately after bolus insulin delivery for both meals and for overall daily infusion after completion of the morning dosing and sampling cycles

VAS pain assessments were completed each day at the breakfast and lunch bolus and after completion of the PK dosing and sampling cycle. Overall, mean pain scores associated with bolus delivery and perception of the daily infusion process were low (Fig. 6); however, self-rated pain was statistically greater for ID compared to SC infusion.

**Fig. 7:**
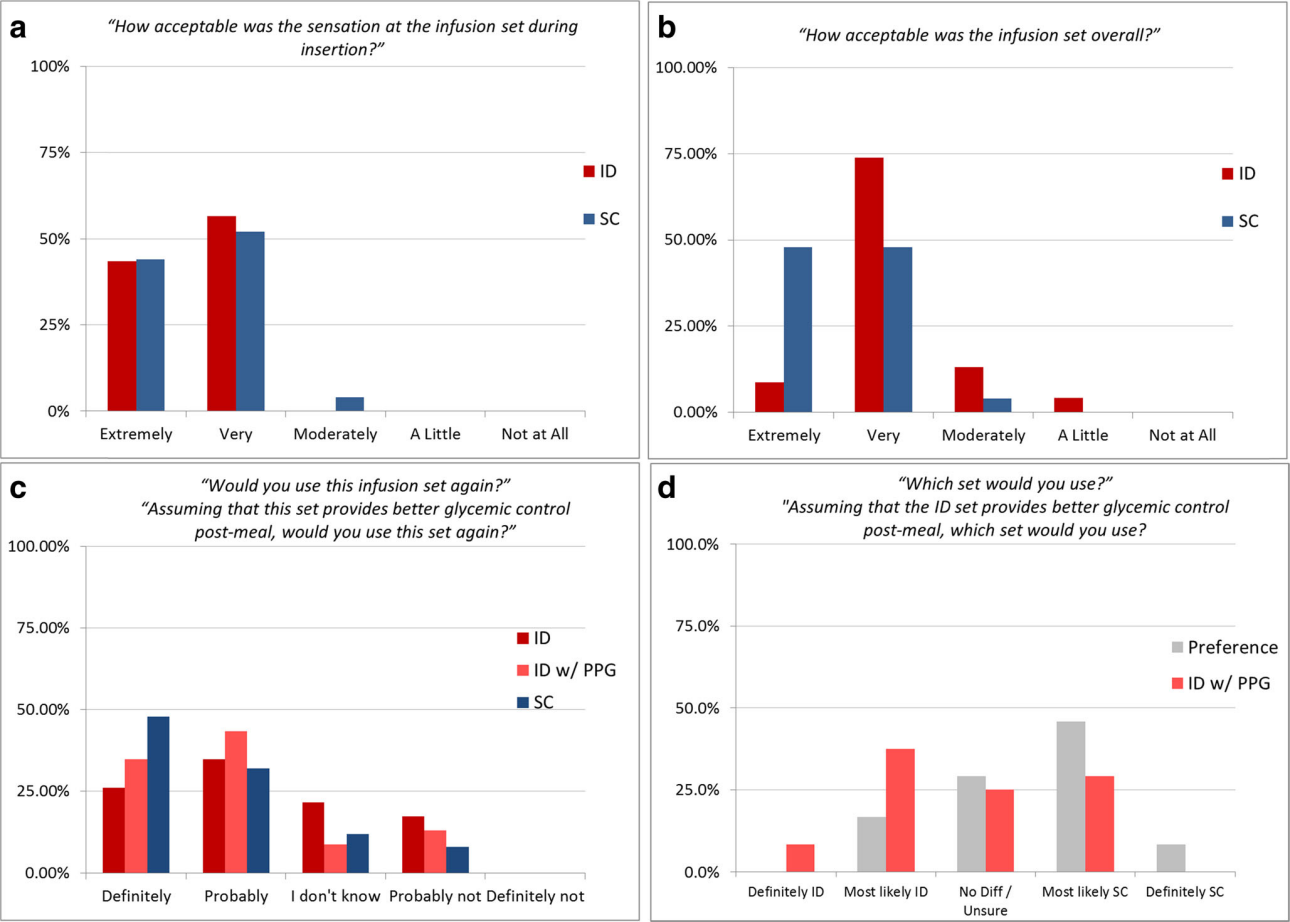
User acceptability questionnaire results (*n* = 24) for acceptability of **a** infusion set insertion, **b** set satisfaction, **c** willingness to use the respective infusion set again, and **d** set preference. Some ID acceptability questions were posed both based on perception alone and within the context of having an increased postprandial glycemic control benefit (ID w/PPG)

Evaluation of the acceptability and preference questionnaires revealed that the insertion of the infusion set was equally acceptable for both ID and SC application, with ratings of 100 and 96 % graded as very or extremely acceptable for each route, respectively. However, acceptability of the infusion set overall favored the SC route, with 96 % reporting as very or extremely acceptable versus 83 % for ID (Fig. 7a, b). When patients were asked about their willingness to use ID infusion sets again, the majority 61 % responded affirmatively (definitely or probably responses; 80 % for SC). Interestingly, when presented with the same question but the potential for better postprandial glycemic (PPG) control using ID administration, this majority increased to 78 % affirmative responses (Fig. 7c). Similarly, user preference to select between device types favored SC (54 % responding definitely or most likely SC vs. 17 % ID; 29 % no difference), but potential PPG benefit shifted preference selection toward ID over SC (46 % responding definitely or most likely ID vs. 29 % SC; 25 % no difference) (Fig. 7d).

## Discussion

The study is the first clinical investigation of extended duration intradermal drug infusion using hollow microneedle technology. Numerous others have examined drug delivery from various microneedle formats, but typically for significantly shorter durations such as bolus vaccine and drug delivery or quick dissolving microneedle patches or coatings. The longest duration microneedle delivery studies have typically involved evaluating passive preclinical delivery for periods up to 24 h [28] after transient application of solid microneedle to disrupt the stratum corneum barrier function. In contrast, this study has demonstrated continuous active fluid transport through a single steel microneedle into the dermal tissue over an extended 3-day period at pharmaceutically relevant insulin delivery rates. Moreover, the investigational microneedle catheter set exhibited multiple attributes including mechanical robustness, the ability to maintain consistency of tissue placement and engagement, and effective fluid path patency to successfully maintain glycemia in T1 diabetes patients over the investigational period. System robustness and efficacy were demonstrated by the effective BG control obtained, a lack of detectable fluid leakage, and effective adhesion throughout the usage period. Additionally, the microneedle infusion set was able to be utilized with commercial insulin pumps originally designed for subcutaneous infusion therapy, with little to no deviation from traditional continuous subcutaneous insulin infusion (CSII) methods. The investigational ID microneedle set demonstrated functional efficacy equivalent to the current recommended 3 day usage period for most commercial CSII set embodiments. This is especially encouraging considering that many current SC CSII sets fail to meet their expected usage lifetime [29].

The investigational microneedle infusion set has features previously described elsewhere [18, 19]. Briefly, the steel microcannula has external and internal dimensions similar to microneedles in other material types [30], a proprietary cannula tip geometry designed to maximize flow reliability, and incorporating proprietary hub surface features to maximize complete insertion. One limitation of the current investigational ID set is the non-optimized adhesive surface, necessitating the use of adjunctive overtaping to maintain long-term adhesion. Although microneedle length dimensions are often cited as <1 mm, the current 1.5-mm microneedle infusion set has been clinically vetted to ensure effective dermal localization and fluid deposition across a range of dosing conditions. Similar steel microcannula and device designs are found in commercially available microneedle bolus injection systems [31]. Other clinical studies have also examined the appropriateness of this length relative to average dermal thicknesses in both normal subjects and diabetics [32, 33].

In addition to establishing microneedle functional feasibility, the primary study objective was to evaluate the long-term impact of intradermal delivery route on previously observed PK time advantages (Tmax) and postprandial glycemic control, including any temporal related changes occurring over the multi-day course of treatment. Recent literature on CSII using traditional SC sets has demonstrated the potential for shift in both PK and PD control parameters with time in as little as 3 days [34–36]. Also, little is known about long-term intradermal delivery kinetics or dermal tissue perfusion and absorption since current extended duration dermal delivery is primarily limited to passive transdermal patches. A statistically significant shorter Tmax was demonstrated for mealtime ID bolus infusions, indicating faster insulin absorption with earlier occurrence of peak serum insulin concentrations. The faster onset is consistent with PK results from prior ID microneedle insulin administration studies. Across days, Tmax was shortened by ~19 min, with time reductions of ~6 and 31 min for T50%_maxrising_ and T50%_maxfalling_, respectively. This was the first demonstrated use of the intradermal insulin aspart analog, where previous delivery studies have used the insulin lispro analog. These results demonstrate the utility of ID microneedle delivery to speed absorption profiles for multiple insulin types. Future work to directly compare ID insulin analogs kinetics in a single patient cohort could be useful.

For all secondary PK endpoints, the difference between the ID and the SC route could be statistically demonstrated with the exception of ΔAUC-INS0-2 and 4 h. With faster ID insulin appearance and disappearance, it was expected that AUC differences observed during the initial rise are reversible during the fall and therefore disappear when analyzing the full excursion. Overall, the analyses of the secondary PK endpoints corresponded with the primary endpoint findings and demonstrated association of ID bolus infusion with more rapid insulin absorption with higher peak concentrations and faster appearance of insulin in systemic circulation, compared to the respective SC infusion. The cross-day Tmax consistency for both ID and SC is in contrast to the above reported CSII examples, where Tmax decreased having faster absorption by day 3. However, in agreement with those references, both routes in the current study exhibited the statistically confirmed trend of increasing overall insulin AUC and Cmax across days. The potential temporal effects on insulin absorption during continuous infusion remain complex and require additional study for both the ID and SC routes. A review [29] of the impact of prolonged site exposure to insulin infusion on metabolic control cites various contradictory examples including reported Tmax reductions [36–38], no changes in PK/PD profiles [39], and average daily BG increases associated with increased set wear duration [40, 41]. The limited and conflicting data on this topic reinforces the need for additional multi-day PK/PD studies designed to elucidate the underlying mechanisms of these observations.

ID microneedle delivery produced ~7 % less intra-subject variability, expressed as percentage coefficient variation (%CV), across meals and days compared to SC administration. This effect was maintained over all three treatment days, with no discernable difference in insulin peak onset time for either the ID or SC study arms. Reductions in insulin absorption variability are encouraging for patients with intensive treatment regimens and for development of predictive closed-loop artificial pancreas (AP) algorithms. The need for both faster and more consistent insulin kinetics has been cited as a critical factor for effective development of closed-loop systems [42, 43].

The postprandial glycemic response, as characterized by the secondary PD endpoints, was significantly less pronounced after ID bolus administration compared to the respective SC bolus. In particular during the first 2 h, the increases in BG-AUC and BG concentrations at different time points were significantly smaller, and the BG maximum as well as the average BG level was lower with ID than the SC bolus. Overall, the analyses of the pharmacodynamic endpoints demonstrated better postprandial glycemic control with ID delivery. A trend of rising blood glucose levels approximately 2 h after the lunch meal and increasing mean peak blood glucose values after day 1 was observed for both administration routes. A review of dosing schedules revealed that additional correction boli were often required after the lunch meal sampling period. This may have contributed to the greater increase in intra-subject variability of some ID glycemic response parameters in contrast to previous published observations. A potential mismatch in the timing of insulin systemic availability and the gastric glucose absorption rate, especially during the more slowly absorbed lunch mixed meal, may also have contributed to this PD variability. In contrast to the day-to-day comparison of PD outcomes, daily serum insulin profiles and Tmax values within routes were similar with daily ΔCmax values typically increasing indicating insulin absorption was not diminished. Further evaluation is required as many interacting factors influence metabolic variability such as differences between injection sites and transient changes insulin sensitivity [44, 45]. With consistent ID time kinetics, it may be feasible to optimize both open and closed-loop prandial insulin infusion to minimize later phase BG excursions with different meal types.

Clinical safety of ID microneedle insulin delivery was consistent with current practice based on the similar frequency and type of reported AEs for each route of administration. Surprisingly, there was no detectable difference in immediate Draize responses at device removal, even though this would have been expected after multi-day continuous infusion into the shallow skin. The delayed onset nature of some observed Draize scores deserves additional investigation to deconvolute potential influences beyond the infusion process alone such as dermal adhesive or insulin reactions.

Overall ID delivery was acceptable per VAS ratings and the patient questionnaire; however, self-rated pain was higher with ID bolus infusion. This result was not unexpected since despite the shorter microneedle length, higher delivery pressures are routinely required for ID bolus delivery due to physical reduction in needle gauge and increased density of the dermal tissue bed compared to the SC space. This increased injection pressure has been demonstrated by ourselves (unpublished data) and others [46] to increase pain perception. This hypothesis is supported by the acceptability data for basal and bolus delivery: ID basal was reported as very or extremely acceptable 96 % of the time (100 % for SC), while ID bolus delivery was very or extremely acceptable only 57 % of the time (88 % for SC). The majority of patients found overall ID microneedle delivery to be acceptable with a willingness to use again. When presented with the prospect of better glucose control, willingness to use ID became equivalent to SC, while preference between sets actually surpassed SC. The challenge of capturing highly subjective responses, such as pain and perception, indicates the need for actual usage within the most clinically relevant context possible. Other mitigating factors for those observational data must also be considered including the small sample numbers, lack of powering for perception endpoints, and additional influences including use of additional adhesive overtaping, the bulky pressure equipment used for delivery monitoring, and the rapid bolus infusion rate of the selected insulin pump. Additional repetitive multi-day evaluations in a more traditional environment are necessary to further characterize the impact of continuous ID insulin infusion on local tissue properties, insulin uptake properties, user acceptance, and overall safety, as well as any potential clinical advantages accruing from the observed PK/PD changes. Device optimization is also necessary to address usage and potentially perception factors.

## Conclusions

This multi-day study is the longest demonstrated use of ID hollow microneedle delivery of insulin in patients with T1DM. Microneedle delivery of the analog insulin aspart was able to achieve both enhanced pharmacokinetic uptake and better prandial glycemic control than SC infusion and maintain these benefits throughout the 3-day testing period. No severe safety concerns or device performance issues were observed throughout the course of the study with overall acceptable device perception and willingness to use among the participants. Current results indicate ID insulin delivery as a viable alternative route providing reduced time for insulin absorption with less intra-subject variability and lower glycemic response and confirming prior clinical findings from shorter duration infusion. Intradermal delivery continues to be a potential candidate for both open and closed-loop insulin delivery systems.

## References

[1] American Diabetes Association (2001). Postprandial blood glucose (consensus statement). Diabetes Care.

[2] Freckmann G, Hagenlocher S, Baumstark A (2007). Continuous glucose profiles in healthy subjects under everyday life conditions and after different meals. J Diabetes Sci Technol (Online).

[3] Cherrington AD (2005). The role of hepatic insulin receptors in the regulation of glucose production. J Clin Invest.

[4] Bolli GB, Di Marchi RD, Park GD, Pramming S, Koivisto VA (1999). Insulin analogues and their potential in the management of diabetes mellitus. Diabetologia.

[5] Home PD (2012). The pharmacokinetics and pharmacodynamics of rapid-acting insulin analogues and their clinical consequences. Diabetes Obes Metab.

[6] Krasner A, Pohl R, Simms P, Pichotta P, Hauser R, De Souza E (2012). A review of a family of ultra-rapid-acting insulins: formulation development. J Diabetes Sci Technol.

[7] Heinemann L, Muchmore DB (2012). Ultrafast-acting insulins: state of the art. J Diabetes Sci Technol.

[8] Cengiz E, Weinzimer SA, Sherr JL (2013). Acceleration of insulin pharmacodynamic profile by a novel insulin infusion site warming device. Pediatr Diabetes.

[9] Fonte P, Araújo F, Reis S, Sarmento B (2013). Oral insulin delivery: how far are we?. J Diabetes Sci Technol.

[10] Rao R, Mahant S, Chhabra L, Nanda S (2014). Transdermal innovations in diabetes management. Curr Diabetes Rev.

[11] Cavaiola TS, Edelman S (2014). Inhaled insulin: a breath of fresh air? A review of inhaled insulin. Clin Ther.

[12] Henkin RI (2010). Inhaled insulin—intrapulmonary, intranasal, and other routes of administration: mechanisms of action. Nutrition.

[13] Pettis RJ, Harvey AJ (2012). Microneedle delivery: clinical studies and emerging medical applications. Ther Deliv.

[14] Hultström M, Roxhed N, Nordquist L (2014). Intradermal insulin delivery a promising future for diabetes management. J Diabetes Sci Technol.

[15] Kim Y-C, Park J-H, Prausnitz MR (2012). Microneedles for drug and vaccine delivery. Adv Drug Deliv Rev.

[16] Donnelly RF, Raj Singh TR, Woolfson AD (2010). Microneedle-based drug delivery systems: microfabrication, drug delivery, and safety. Drug Deliv.

[17] Harvey AJ, Kaestner SA, Sutter DE, Harvey NG, Mikszta JA, Pettis RJ (2011). Microneedle-based intradermal delivery enables rapid lymphatic uptake and distribution of protein drugs. Pharm Res.

[18] Pettis RJ (2011). Microneedle-based intradermal versus subcutaneous administration of regular human insulin or insulin lispro: pharmacokinetics and postprandial glycemic excursions in patients with type 1 diabetes. Diabetes Technol Ther.

[19] McVey E (2012). Pharmacokinetics and postprandial glycemic excursions following insulin lispro delivered by intradermal microneedle or subcutaneous infusion. J Diabetes Sci Technol.

[20] Meric-Bernstam F (2014). Toward nodal staging of axillary lymph node basins through intradermal administration of fluorescent imaging agents. Biomed Optics Express.

[21] Milewski M (2015). Analysis of the absorption kinetics of macromolecules following intradermal and subcutaneous administration. Eur J Pharm Biopharm.

[22] McVey EA, Keith SC, Sutter DE, Judge K, Herr J, Pettis RJ. Proof of reliability of intradermal devices under extended wear basal/bolus infusion conditions. Poster abstract EASD Barcelona 2013; data submitted for publication.

[23] Heinemann L, et al. Intra-dermal insulin lispro application with a new microneedle delivery system led to a substantially more rapid insulin absorption than subcutaneous injection. Diabetologia. Vol. 49. 233 Spring Street, New York, Ny 10013 USA: SPRINGER; 2006.

[24] Gupta J, Felner EI, Prausnitz MR (2009). Minimally invasive insulin delivery in subjects with type 1 diabetes using hollow microneedles. Diabetes Technol Ther.

[25] Gupta J, Felner EI, Prausnitz MR (2011). Rapid pharmacokinetics of intradermal insulin administered using microneedles in type 1 diabetes subjects. Diabetes Technol Ther.

[26] Pettis RJ (2011). Intradermal microneedle delivery of insulin lispro achieves faster insulin absorption and insulin action than subcutaneous injection. Diabetes Technol Ther.

[27] Norman JJ (2013). Faster pharmacokinetics and increased patient acceptance of intradermal insulin delivery using a single hollow microneedle in children and adolescents with type 1 diabetes. Pediatr Diabetes.

[28] Mohammed YH, Yamada M, Lin LL, Grice JE, Roberts MS, Raphael AP (2014). Microneedle enhanced delivery of cosmeceutically relevant peptides inhuman skin. PLoS ONE.

[29] Heinemann L, Krinelke L (2012). Insulin infusion set: the Achilles heel of continuous subcutaneous insulin infusion. J Diabetes Sci Technol.

[30] Gill HS, Denson D, Burris B, Prausnitz MR (2008). Effect of microneedle design on pain in human subjects. Clin J Pain.

[31] Laurent PE, Bonnet S, Alchas P (2007). Evaluation of the clinical performance of a new intradermal vaccine administration technique and associated delivery system. Vaccine.

[32] Laurent A, Mistretta F, Bottigioli D, Dahel K, Goujon C (2007). Echographic measurement of skin thickness in adults by high frequency ultrasound to assess the appropriate microneedle length for intradermal delivery of vaccines. Vaccine.

[33] Gibney MA, Arce CH, Byron KJ (2010). Skin and subcutaneous adipose layer thickness in adults with diabetes at sites used for insulin injections: implications for needle length recommendations. Curr Med Res Opin.

[34] Luijf YM, Arnolds S, Avogaro A (2013). Patch pump versus conventional pump: postprandial glycemic excursions and the influence of wear time. Diabetes Technol Ther.

[35] T1D exchange evaluates correlation between infusion set duration and FBG. (2014, July 7). Retrieved May 8, 2015, from https://t1dexchange.org/pages/t1d-exchange-evaluates-correlation-between-infusion-set-duration-and-fbg/.

[36] Clausen TS, Kaastrup P, Stallknecht B (2009). Effect of insulin catheter wear-time on subcutaneous adipose tissue blood flow and insulin absorption in humans. Diabetes Technol Ther.

[37] Liu D (1991). Insulin absorption is faster when keeping the infusion site in use for three days during continuous subcutaneous insulin infusion. Diabetes Res Clin Pract.

[38] Swan KL (2009). Effect of age of infusion site and type of rapid-acting analog on pharmacodynamic parameters of insulin boluses in youth with type 1 diabetes receiving insulin pump therapy. Diabetes Care.

[39] Olsson PO, Arnqvist H, Asplund J (1993). No pharmacokinetic effect of retaining the infusion site up to four days during continuous subcutaneous insulin infusion therapy. Diabet Med.

[40] Thethi TK (2010). Consequences of delayed pump infusion line change in patients with type 1 diabetes mellitus treated with continuous subcutaneous insulin infusion. J Diabetes Complicat.

[41] Schmid V (2010). Pilot study for assessment of optimal frequency for changing catheters in insulin pump therapy—trouble starts on day 3. J Diabetes Sci Technol.

[42] Cobelli C, Renard E, Kovatchev B (2011). Artificial pancreas: past, present, future. Diabetes.

[43] Elleri D, Dunger DB, Hovorka R (2011). Closed-loop insulin delivery for treatment of type 1 diabetes. BMC Med.

[44] Heinemann L (2002). Variability of insulin absorption and insulin action. Diabetes Technol Ther.

[45] Kildegaard J, Christensen TF, Hejlesen OK (2009). Sources of glycemic variability—what type of technology is needed?. J Diabetes Sci Technol.

[46] Gupta J, Park SS, Bondy B, Felner EI, Prausnitz MR (2011). Infusion pressure and pain during microneedle injection into skin of human subjects. Biomaterials.

